# Association of dietary approaches to stop hypertension, genetic predisposition, and the risk of musculoskeletal disorders: a prospective cohort study

**DOI:** 10.1186/s12891-026-10213-4

**Published:** 2026-07-20

**Authors:** Bin Yang, Di Zhang, Xin Song, Xiaofeng Ma, Sirui Zheng, Rong Xiang, Xunying Zhao, Yang Qu, Tao Han, Ting Yu, Jinyu Zhou, Maoyao Xia, Yangdan Zhong, Zihao Li, Ye Ju, Yuqi Pang, Hongxia Xia, Ben Zhang, Mengyu Fan, Xia Jiang

**Affiliations:** 1 Department of Nutrition and Food Hygiene, West China School of Public Health and West China Fourth Hospital, Sichuan University, Chengdu, Sichuan China; 2 Department of Epidemiology and Biostatistics, , Institute of Systems Epidemiology, and West China-PUMC C. C. Chen Institute of Health, West China School of Public Health and West China Fourth Hospital, Sichuan University, Chengdu, Sichuan China; 3 Department of Clinical Neuroscience, Karolinska Institute, Stockholm, Sweden

**Keywords:** Dietary Approaches to Stop Hypertension, Musculoskeletal Diseases, Polygenic risk score, Cohort study

## Abstract

**Objective:**

To investigate the associations of the Dietary Approaches to Stop Hypertension (DASH) diet with the incidence of overall and specific musculoskeletal (MSK) disorders, taking genetic predispositions into account.

**Methods:**

Study populations included 115,692 participants. DASH diet scores were calculated using the Oxford WebQ dietary questionnaire, and poly-genetic risk scores (PRS) were calculated summing weighted risk alleles. Cox regressions were performed to evaluate the associations between DASH diet, PRS, and incidence of MSK disorders. The primary outcome was overall disease defined as any of MSK disorders. The secondary outcomes were five major specific conditions, including osteoarthritis, rheumatoid arthritis, gout, low back pain, and neck pain.

**Results:**

During a median follow-up period of 11.64 years, 33,230 incident cases of overall MSK disorders were observed. Each 5-point increment in the DASH diet score was associated with a modest 2–4% reduced risk of overall MSK disorders, consistent across three models (Model 1: HR = 0.96, 95%CIs = 0.95–0.97; Model 2: HR = 0.98, 95%CIs = 0.97–0.99; Model 3: HR = 0.98, 95%CIs = 0.97–0.99) as well as different genetic risk groups (low: HR = 0.95, 95%CIs = 0.87–1.03; moderate: HR = 0.98, 95%CIs = 0.94–1.03; high: HR = 0.95, 95%CIs = 0.87–1.03). No gene-diet interaction was identified. When extending to the five specific MSK disorders, a similar pattern of results was observed, except for RA and gout.

**Conclusions:**

Adherence to DASH diet is associated with a modestly reduced risk of overall MSK disorders in European populations. In disease-specific analyses, protective associations were observed for OA, low back pain, and neck pain, whereas associations with RA and gout were less consistent. The protective association is broadly consistent across most genetic risk strata, although a potential interaction was noted for RA.

**Supplementary Information:**

The online version contains supplementary material available at https://doi.org/10.1186/s12891-026-10213-4.

## Introduction

Musculoskeletal (MSK) disorders, characterized by inflammation, degeneration, chronic joint pain and damage [1], are the leading cause of disability and premature mortality worldwide [2]. Among these disorders, osteoarthritis (OA), rheumatoid arthritis (RA), gout, low back pain, and neck pain contribute the most to burden of disease [3]. With an accelerated global aging, the prevalence of MSK disorders is expected to increase significantly [4], emphasizing an urgent need to develop effective prevention strategies.

The intricate etiology of MSK disorders involve genetic predisposition, environmental factors, and their complex interplay [5]. Diet has gained increasing attention in recent years, as it is a common and actively modifiable factor in daily life. The DASH (Dietary Approaches to Stop Hypertension) diet which advocates the consumption of whole grains, vegetables, fruits, low-fat dairy products, legumes, and nuts [6] has been shown not only to improve metabolic profile [7, 8], but also promote bone health through its anti-inflammatory and anti-oxidant properties [9]. Indeed, two previous studies found that adherence to DASH diet was associated with a lower risk of OA (OR = 0.78, 95%CIs = 0.60–1.00) [10] and gout (HR = 0.68, 95%CIs = 0.57–0.80) [11] in the US populations. However, epidemiological evidence remains limited – the only two available studies so far both focused on specific MSK disorders, were of small sample sizes and consisted predominantly a single sex. There is a lack of large-scale, well-designed prospective cohort study to systematically examine the role of DASH diet on overall risk of MSK disorders, particularly in non-US populations.

The impact of DASH diet on MSK disorders may vary according to an individual’s genetic predisposition [12]. Genome-wide association studies (GWAS) have identified multiple single nucleotide polymorphisms (SNPs) linked to specific MSK disorders [13–15], enabling the calculation of polygenic risk score (PRS). Recent advances in genetic risk prediction have led to the development of metaPRS – a multi-PRS approach that combines multiple trait-specific PRSs into a single metric [16] – which significantly increases the predictive accuracy for various complex diseases [17]. However, research on the interplay between genetic risk and dietary intervention in MSK disorders remains limited. Only one study has explored the combined effect of gene-DASH diet on the risk of gout [18]. Whether individuals with a high genetic susceptibility to overall or other specific MSK disorders could benefit from adhering to DASH diet is still unknown.

Utilizing large-scale population-based data from the UK Biobank (UKB), which includes comprehensive questionnaires and genetic data, this study aims to: 1) investigate the associations of adherence to DASH diet with the incidence of overall MSK disorders; 2) examine potential interactions between DASH diet adherence and genetic predisposition in modulating MSK disorder risk. Additionally, we extended the analyses to five specific MSK disorders.

## Materials and methods

### Study population

Our research data were derived from UKB, a large-scale prospective cohort comprising over 500,000 participants aged 40–69 years. Participants were recruited across 22 assessment centers in England, Scotland, and Wales between 2006 and 2010. UKB collected data from participants through questionnaires, verbal interviews, physical measurements and biological sampling, including demographics, lifestyle habits and health information [19]. Ethical approval was received from the North West Multi-Center Research Ethics Committee (11/NW/0382) and every participant gave their written informed consent. The present research was conducted under application number 99713.

**Fig. 1:**
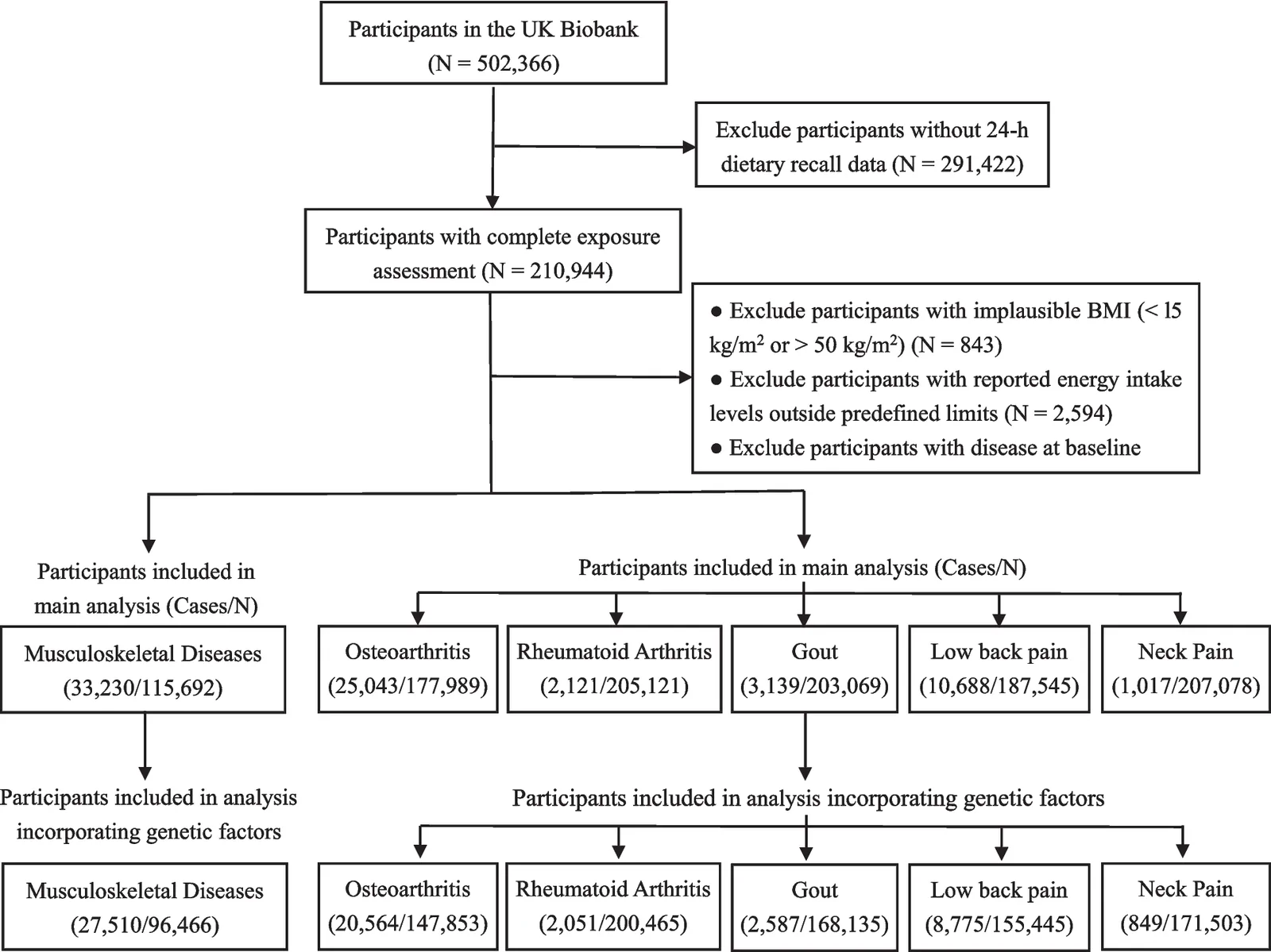
.

We excluded participants who lacked 24-h dietary recall data, had implausible BMI values (< 15 or > 50 kg/m^2^), reported extreme energy intake (female: < 500 or > 3500 kcal/d, male: < 800 or > 4200 kcal/d), or had pre-existing MSK disorders at baseline. For overall MSK disorders, participants with a history of any MSK disorder at baseline were excluded. For each specific outcome, participants were excluded only if they had a diagnosis of that specific condition at baseline. After exclusions, the primary analysis on overall MSK disorders included 115,692. For genetic analyses, 96,466 individuals with complete genotyping data were retained and further divided into a training set (n = 28,940) for metaPRS derivation and a testing set (n = 67,526) for evaluating metaPRS performance. The numbers of participants for primary analysis of OA, RA, gout, low back pain and neck pain were 177,989, 205,121, 203,069, 187,545 and 207,078, respectively and for genetic analysis were 147,853, 200,465, 168,135, 155,445 and 171,503, respectively. The flowchart for participant selection was shown in Fig. 1.

### Assessment of DASH diet

Dietary information in UKB was assessed through five rounds of 24-h dietary recall surveys conducted between April 2009 and June 2012 using the Oxford WebQ dietary questionnaire [20]. Participants completed the recall one to five times (average: 2.2 times), with the distribution as follows: one assessment (38%), two assessments (23%), three assessments (21%), four assessments (15%), and five assessments (3%), and a mean dietary intake value was computed for those with multiple assessments. Adherence to the DASH diet was quantified using the validated scoring system developed by Fung et al. [21], which evaluates intake across 8 key food groups: fruits, vegetables, whole grains, nuts and legumes, low-fat dairy products, red and processed meats, sweetened beverages and sodium. For each food group, participants were classified into quintiles according to their intake ranking. Beneficial dietary components (fruits, vegetables, whole grains, nuts and legumes and low-fat dairy products) were assigned positive scores ranging from 1 (lowest intake quintile) to 5 (highest intake quintile). Conversely, less healthy components (red/processed meats, sweetened beverages, and sodium) were inversely scored (1 for highest intake quintile and 5 for lowest intake quintile) [22]. The individual component scores were summed to derive a total DASH diet for each participant, with possible scores ranging from 8 to 40 points. Higher scores indicate greater adherence to the DASH dietary pattern. Detailed information of DASH diet scoring methodology is provided in Supplementary Table S1.

### Assessment of the outcome

The primary outcome was overall MSK disorders (not limited to the five major conditions analyzed in secondary analyses). The secondary outcomes included the incidence of five specific, high-burden MSK conditions, including RA, OA, gout, low back pain, and neck pain. We used any hospital admission with an International Classification of Diseases, Ninth Revision (ICD-9) or Tenth Revision (ICD-10) codes to identify MSK disorders. Supplementary Table S2 provides detailed information on the ICD-9 and ICD-10 codes. Baseline was defined as the date when participants first completed the 24-h dietary recall survey. Follow-up duration was calculated from baseline until the diagnosis of a MSK disorder, death, loss to follow-up, or the censoring date, whichever came first.

### Assessment of the genetic risk score

For RA, we utilized the precomputed “Standard PRS” from the UK Biobank PRS Release, which has shown satisfactory predictive performance [23]. We used PLINK to calculate the PRSs for the remaining four MSK disorders (OA, gout, low back pain, and neck pain). The genotyping, quality control, and imputation data and more detailed information of UKB genetic data have been described in detail previously [24]. We extracted significant SNPs associated with each MSK disorder according to previously published well-conducted GWASs. We excluded SNPs with a minor allele frequency < 0.005 or an imputation quality score < 0.4. The information of candidate SNPs used to calculated trait-specific PRS is presented in Supplementary Table S3-6.

A metaPRS was subsequently generated to comprehensively quantify the genetic susceptibility to overall MSK disorders by integrating trait-specific PRSs. First, each PRS was standardized to zero mean and unit SD. The dataset was then randomly split into training (70%) and testing sets (30%). In the training set, an elastic-net logistic regression with tenfold cross-validation was performed via the R package “glmnet” to examine the association between the trait-specific PRSs and the overall MSK disorders, adjusting for age, sex, genetic arrays, and the top 10 genetic principal components [16]. Finally, the metaPRS was calculated in the test set using the adjusted coefficients for each PRS derived from this model as weights via a weighted sum.

$$\mathrm{P}\mathrm{R}\mathrm{S}\mathrm{i}\mathrm{m}\mathrm{e}\mathrm{t}\mathrm{a}=\frac{{\alpha}_{1}{PRS}_{i1}+{\alpha}_{2}{PRS}_{i2}+\dots {+\alpha }_{5}{PRS}_{i5}}{{\alpha}_{1}+\dots +{\alpha}_{5}}$$

where ${PRS}_{i1}$,…, and ${PRS}_{i5}$ are the five zero mean and unit variance standardized PRSs for *ith* the individual; ${\alpha}_{1}$,…, and ${\alpha}_{5}$ are the coefficients for each of the five PRSs. Participants were categorized into low (the bottom quintile), moderate (quintiles 2–4) and high (the top quintile) genetic risk groups according to the distribution of their trait-specific PRSs or metaPRS.

### Covariates

The covariates included age (continuous), sex (male or female), and ethnicity (white or others), education level (college/university degree or others), socioeconomic status, BMI (continuous), physical activity (low, moderate, or high), smoking status (current, previous, or never), alcohol consumption (current, previous, or never), sleep duration (< 7 h, 7–8 h, or > 8 h), energy intake (continuous), multivitamin use (yes or no), and the medication use of antihyperlipidemic, antihypertension and antidiabetic (yes or no). Socioeconomic status was measured through the Townsend Deprivation Index (TDI), with higher scores indicating higher levels of deprivation [25, 26].

### Statistical analysis

Baseline characteristics were presented as mean (SD) for continuous variables and n (%) for categorical variables, and differences across quintiles of the DASH diet score were tested using one-way ANOVA for continuous variables and chi-square test for categorical variables. For missing data, we employed median imputation for continuous variables and created a separate “other” category for categorical variables.

The DASH diet score was evaluated both as a continuous variable (scaled in five-point increments) and as a categorical variable (divided into quintiles with the lowest quintile as the reference group). We used the Cox proportional hazards model to assess the association of DASH diet score and PRSs with the incidence risk of MSK disorders. Model 1 was adjusted for age, sex and ethnicity. Model 2 additionally adjusted for Townsend deprivation Index (TDI), education level, BMI, physical activity, smoking status, alcohol consumption, sleep duration and energy intake. Model 3 additionally adjusted for multivitamin use, medication use of antihyperlipidemic, antihypertension and antidiabetic. For genetic analyses involving PRS or metaPRS, we further adjusted for the first 10 genetic principal components and genotyping batch. The Schoenfeld residuals method was used to test the proportional-hazard assumption, and no violations were detected.

In addition, we examined the joint association of DASH diet and PRS/metaPRS with MSK disorders. The reference group comprised the highest-risk participants, defined as those with both the lowest DASH diet score and the highest PRS/metaPRS. We assessed additive interactions by calculating three key metrics with 95% confidence intervals: relative excess risk due to interaction (RERI), attributable proportion due to interaction (AP), and synergy index (S). For multiplicative interactions, we conducted likelihood ratio tests comparing models with and without the multiplicative interaction term.

Subgroup analysis were conducted by age (< 60 or ≥ 60), sex (male or female), ethnicity (white or others), TDI (< 0 or ≥ 0), education level (college/university degree or others), BMI (< 30 or ≥ 30), sleep duration (7–8 h or others), physical activity (low, moderate, or high), smoking status (current, previous, or never), alcohol consumption (current, previous, or never).

Sensitivity analyses were conducted to verify the robustness of results. First, we re-ran the main analysis based on dataset imputed through multiple imputation. Second, we excluded individuals diagnosed with MSK within the first two years of follow-up to mitigate the potential impact of reverse causality. Third, we excluded participants with less than two dietary assessments. Fourth, we further adjusted for vitamin D. Fifth, we further adjusted for occupational physical load. All analyses were completed using R software (version 4.3.0). A two-sided p-value of less than 0.05 was considered statistically significant.

## Results

### Population Characteristics

The baseline characteristics of participants involved in the analysis of overall MSK disorders are provided in Table 1, and the baseline characteristics of participants involved in studies of each five specific MSK disorders are provided in Supplementary Tables S7-11. In general, participants with higher DASH diet score tended to be older, female, have a university degree and better socioeconomic status, and exhibit more favorable health profiles, including lower smoking rates, lower alcohol consumption, lower calorie intake, higher physical activity levels, and longer sleep duration.

### Association of DASH diet with the risk of MSK disorders

During a median follow-up of 11.64 years, we observed 33,230 incident cases of any MSK disorders. As shown in Table 2, each 5-point increment in the DASH diet score was associated with a 2–4% reduced risk of overall MSK disorders across all three models (Model 1: HR = 0.96, 95%CIs = 0.95–0.97; Model 2: HR = 0.98, 95%CIs = 0.97–0.99; Model 3: HR = 0.98, 95%CIs = 0.97–0.99). When examining DASH adherence by quintiles, individuals in the highest quintile (Q5) showed a 3–9% lower risk of incident overall MSK disorders compared to those in the lowest quintile (Q1), with statistically significant associations in Model 1 (HR = 0.91, 95%CIs = 0.88–0.94) as well as in Model 2 (HR = 0.97, 95%CIs = 0.93–0.99), and a borderline significant association in model 3 (HR = 0.97, 95%CIs = 0.94–1.00).

Such a protective association was observed in the analysis with each specific MSK disorders including OA (DASH diet score as continuous variable: HR = 0.98, 95%CIs = 0.96–0.99; as categorical variable: HR = 0.95, 95%CIs = 0.91–0.98), gout (continuous variable: HR = 0.94, 95%CIs = 0.90–0.98; categorical variable: HR = 0.89, 95%CIs = 0.82–0.96), low back pain (continuous variable: HR = 0.96, 95%CIs = 0.94–0.98; categorical variable: HR = 0.91, 95%CIs = 0.86–0.96) and neck pain (continuous variable: HR = 0.92, 95%CIs = 0.85–0.99; categorical variable: HR = 0.80, 95%CIs = 0.66–0.97), despite a non-significant association in RA (continuous variable: HR = 0.96, 95%CIs = 0.91–1.01; categorical variable: HR = 0.92, 95%CIs = 0.81–1.05).

### DASH diet, genetic risk and the risk of MSK disorders

Compared to participants with low genetic risk, those with high genetic risk showed significantly greater susceptibility to overall MSK disorders (HR = 1.14, 95%CIs = 1.10–1.18). This pattern of elevated genetic risk was consistently observed across all specific MSK conditions examined, except for low back pain and neck pain, where the associations did not reach statistical significance (Supplementary Table S12).

**Fig. 2:**
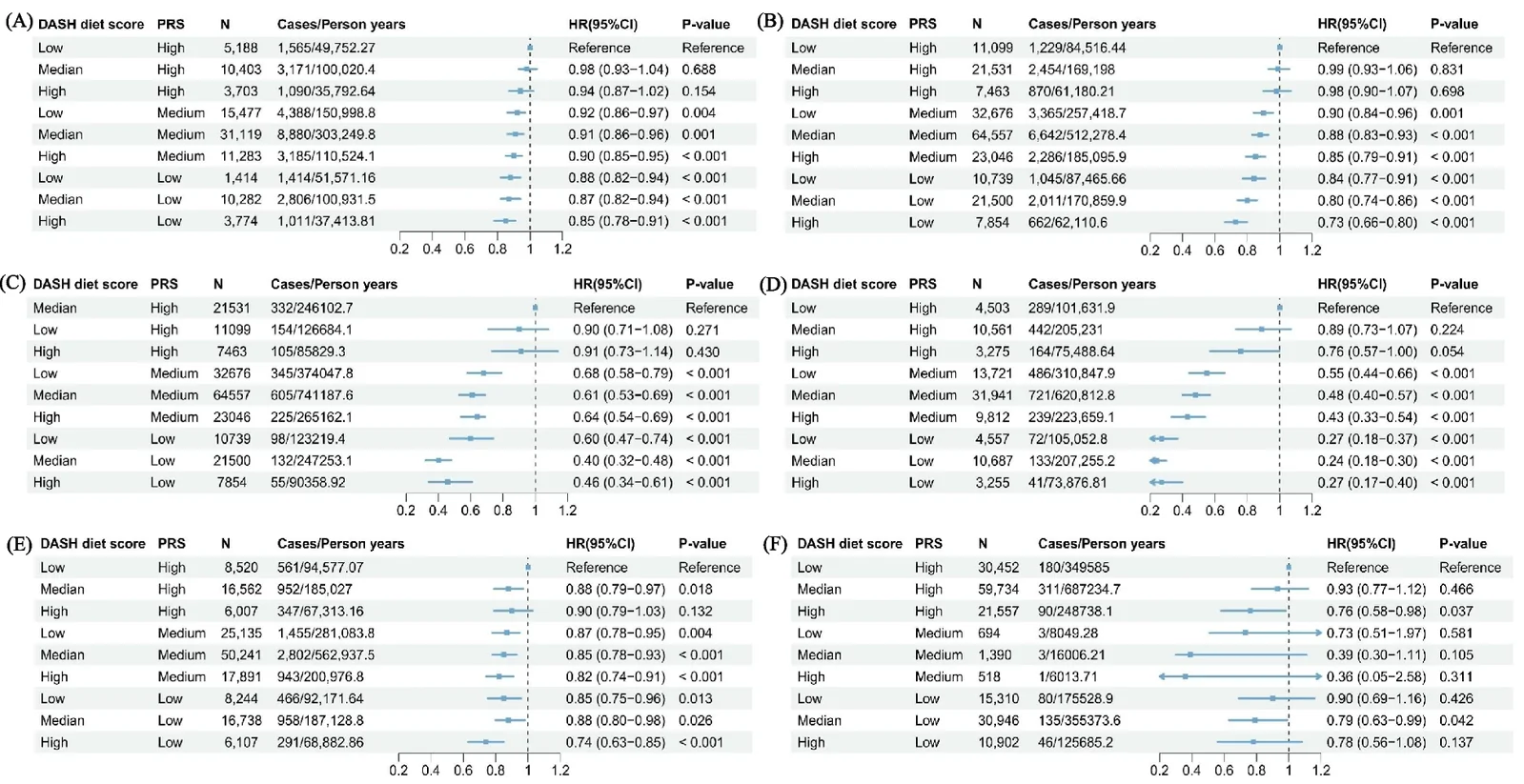
.

In joint analysis, participants with a low metaPRS and a high DASH diet score showed the lowest risk of overall MSK disorders (HR = 0.85, 95%CIs = 0.78–0.91) compared to those with a high metaPRS and a low DASH diet score (Fig. 2). Unfortunately, adherence to DASH diet did not appear to modify the genetic risk of overall MSK disorders. For example, participants with a moderate metaPRS and a low DASH diet score (HR = 0.92, 95%CIs = 0.86–0.97) showed a comparable/slightly lower disease risk to those with a high metaPRS but a high DASH diet score (HR = 0.94, 95%CIs = 0.87–1.02). Similar pattern of results was observed in each specific MSK disorder. Additive interaction between DASH diet score and PRS on incident RA were identified as the RERI was found to be 0.25 (95%CIs = 0.03–0.47) (Supplementary Table S13).

In stratified analysis by genetic risk categories, higher DASH diet adherence was consistently associated with a lower risk of overall MSK disorders across all genetic risk groups, albeit with larger statistical uncertainties possibly due to a reduced power (low metaPRS: HR = 0.95, 95%CIs = 0.87–1.03; moderate metaPRS: HR = 0.98, 95%CIs = 0.94–1.03; high metaPRS: HR = 0.95, 95%CIs = 0.87–1.03). No significant interaction between DASH diet and polygenic risk for overall MSK disorders was found (*P*_interaction_ > 0.05). This pattern was generally consistent across specific MSK conditions, with one notable exception – for RA, we identified a statistically significant multiplicative interaction between DASH diet and genetic risk (*P*_interaction_ = 0.042) (Supplementary Fig. 1), suggesting that dietary effects may differ by genetic susceptibility for this autoimmune condition.

### Subgroup and sensitivity analyses

**Fig. 3:**
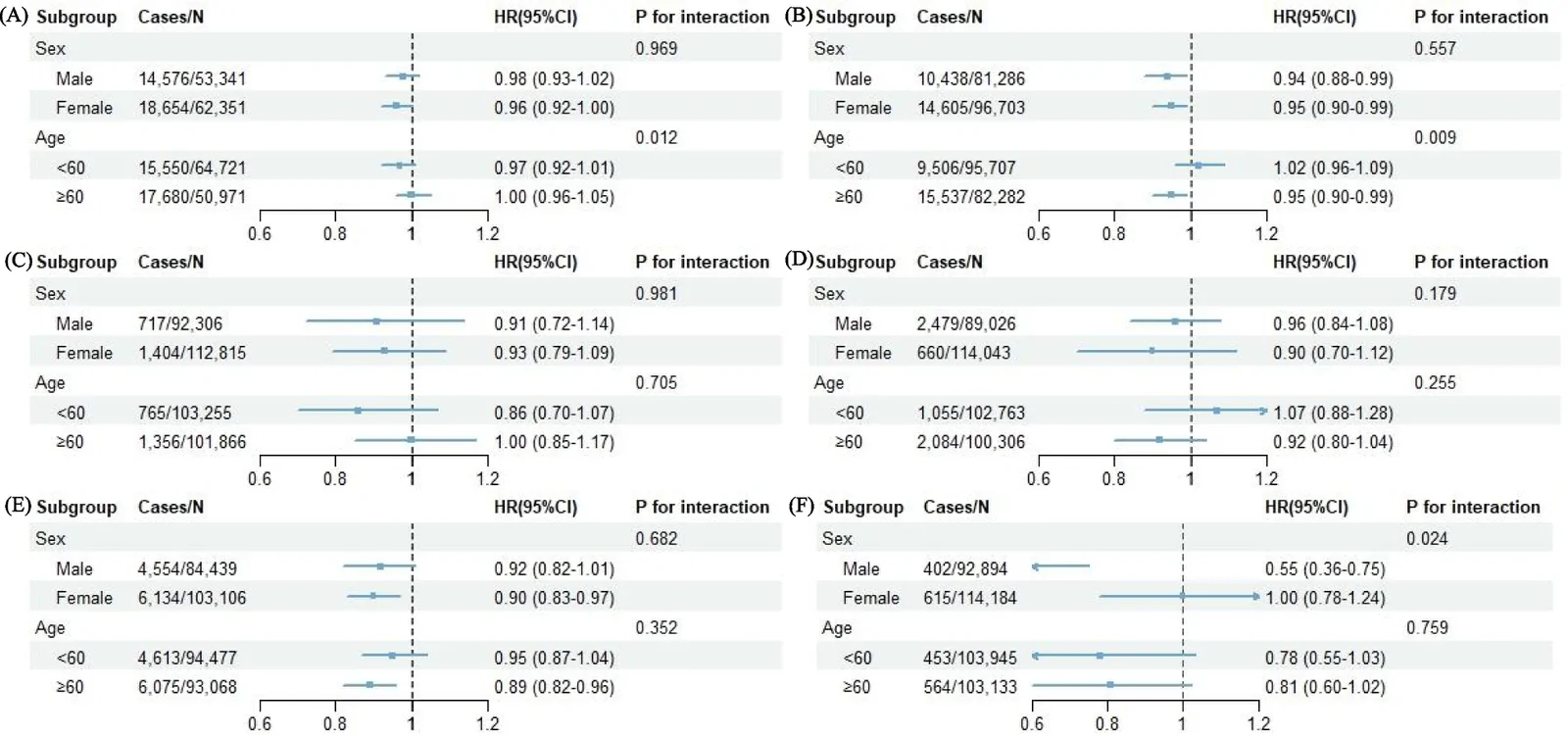
.

Our stratified analyses revealed that while the DASH diet showed protective associations against MSK disorders across all subgroups, the magnitude of benefit varied significantly by age and sex. For overall MSK disorders, the protective association of DASH diet was stronger in younger participants (< 60 years; *P*_interaction_ < 0.05). For OA specifically, older individuals (*≥* 60 years) derived greater benefit (*P*_interaction_ < 0.05). Sex-specific analyses showed the protective association against neck pain was particularly pronounced among male participants (*P*_interaction_ < 0.05) (Fig. 3) (Supplementary Fig. 2).

The associations of DASH diet with the risk of incident overall and specific MSK disorders remained largely consistent in sensitivity analysis where multiple imputation was conducted to the dataset, individuals diagnosed within the first two years of follow-up were excluded, individuals with less than two dietary assessments were excluded, vitamin D was further adjusted, and occupational physical load was further adjusted. (Supplementary Table S14).

## Discussion

In this large-scale prospective cohort study, we systematically examined the associations of the DASH diet and musculoskeletal health, while also investigating potential interactions with genetic predisposition. We observed a modest inverse association between DASH diet adherence and the risk of overall MSK disorders. Notably, this protective association remained consistent across most genetic risk strata, with the exception of a potential interaction observed for RA. When examining five specific MSK disorders, the associations were consistent across most conditions, with protective associations observed for OA, low back pain, and neck pain, whereas findings for RA and gout were less consistent. This heterogeneity across disease subtypes suggested that the protective association of DASH diet was broadly applicable across most musculoskeletal conditions, though the disease-specific results should be considered the primary evidence for etiological inference.

Previous epidemiological studies have established associations between the DASH diet and specific MSK disorders, including a 22% reduced risk of OA (OR = 0.78, 95%CI = 0.60–1.00) in US populations [10] and a 32% reduced risk of gout in men (HR = 0.68, 95%CI = 0.57–0.80) [11]. While these findings demonstrated benefits for individual conditions, they did not examine overall MSK disorders risk or consider genetic susceptibility, and were limited to US populations. Our study extends this evidence by showing that DASH diet adherence is associated with reduced incidence of both overall and specific MSK disorders in European populations. Furthermore, for the first time, we confirm that the protective association of DASH diet persists across all genetic risk strata. This represents a key advancement, as it integrates genetic backgrounds with dietary factors to understand disease etiology. Our findings thus not only confirm previous disease-specific associations but also provide novel insights into the potential of the DASH diet for MSK prevention, particularly in European populations.

In our study, each 5‑point increment in the DASH diet score was associated with a 2–4% relative risk reduction for overall MSK disorders. While this effect size is modest at the individual level, it may translate into a meaningful population health impact given the high prevalence of musculoskeletal disorders, which affect approximately 1.71 billion people globally [27]. The corresponding absolute risk reduction over the 11.64‑year follow‑up was approximately 0.9 percentage points, with a number needed to treat of approximately 116. These findings provide supportive evidence for promoting the DASH diet as part of a population‑based strategy for musculoskeletal health, although the clinical significance at the individual level should not be overstated. While direct mechanistic studies linking DASH diet to musculoskeletal health remain limited, several biologically plausible pathways may explain these protective associations. The abundant micronutrient profile in DASH diet, particularly its rich supply of various vitamins and antioxidants, may mitigate musculoskeletal degeneration by reducing oxidative stress and decreasing neuroinflammation [28, 29]. Additionally, the emphasis on adequate protein intake in DASH diet supports critical musculoskeletal maintenance processes, including bone matrix formation and skeletal muscle maintenance [30]. Emerging evidence also suggests that the DASH diet promotes a beneficial gut microbiome profile [31], which may indirectly influence musculoskeletal health through enhanced nutrient absorption and reduced systemic inflammation [32].

Our subgroup analyses revealed important variations in the protective associations of DASH diet across different populations. Notably, younger individuals exhibited stronger benefits for overall MSK disorders, potentially attributable to their greater anabolic sensitivity [33] and enhanced gut microbiota plasticity [34]. Interestingly, this pattern was reversed for OA, where older adults showed greater protection, likely related to fundamental differences in OA pathophysiology [35]. The magnitude of association between DASH diet and neck pain was greater among males, possibly related to differences in occupational exposure and muscle loading [36]. Importantly, despite these variations, we consistently observed protective associations of DASH diet across all subgroups. These findings suggest the fundamental protective role of this dietary pattern appears broadly generalizable across diverse populations, supporting its implementation as a foundational component of public health approaches to musculoskeletal health. In addition, the composite MSK outcome comprises conditions with distinct etiologies, which may obscure condition-specific biological pathways. Therefore, the disease-specific analyses should be considered complementary evidence for etiological inference, while the composite finding provides a broader population-level perspective.

Our study provides evidence on the interaction between genetic predisposition and the DASH diet on incident of overall MSK disorders. While genetic susceptibility and gene-diet interactions play a considerable role in the incidence of many complex diseases [37], our study did not detect statistically significant interactions, except for RA. However, the fact that protective associations were observed across most genetic risk strata, with the exception of RA, further supports that DASH diet may have a potential role in musculoskeletal health across the general population, without the need for complex and costly genetic risk stratification. Notably, we observed a potential gene-diet interaction for RA. The multiplicative interaction was statistically significant (P for interaction = 0.042), with the stratified analyses showing that the protective association of DASH diet was more pronounced among individuals with lower genetic susceptibility to RA. The relative excess risk due to interaction (RERI) was 0.25, indicating a positive additive interaction. The combined effect of high genetic risk and low DASH adherence on RA risk exceeded the sum of their individual effects on the absolute risk scale. Biologically, given RA’s distinct HLA‑driven autoimmune pathophysiology, dietary factors with anti‑inflammatory properties may modulate immune pathways [38, 39]. However, the modest case number and the relatively wide confidence intervals warrant cautious interpretation. The findings should therefore be considered hypothesis‑generating and require replication in independent cohorts with larger sample sizes.

To our knowledge, this is the first large-scale prospective cohort study to comprehensively evaluate associations of DASH diet with musculoskeletal health while considering the effect of genetic susceptibility. However, several limitations exist. First, dietary intake was assessed using a self-reported 24-h food recall, which may be subject to recall bias despite its practicality in large-scale epidemiological studies. Second, despite comprehensive adjustment for sociodemographic, lifestyle, and health-related factors, residual confounding cannot be completely ruled out. While we adjusted for medication use as a proxy for certain comorbidities, we lacked a comprehensive comorbidity index (e.g., Charlson index) for full adjustment. Third, the predictive power of the PRS for neck pain was very limited (AUC = 0.519, 95%CIs = 0.501–0.537), which is consistent with the relatively small number of SNPs available for PRS construction, highlighting the need for future larger-scale GWAS to identify additional genetic variants. Furthermore, constructing a metaPRS based on the five major diseases may not fully capture the overall genetic susceptibility to MSK disorders and may have attenuated the detection of gene-diet interactions. Fourth, case ascertainment through inpatient records potentially missed milder cases managed in outpatient or primary care settings, particularly for pain-related conditions. This may introduce misclassification and would likely attenuate the true protective associations, suggesting that our observed associations may be conservative. Future studies incorporating primary care linkage data would further strengthen the evidence. Fifth, healthy volunteer bias may lead to underestimation of the observed associations. Additionally, the study population consisted primarily of individuals of European descent, limiting generalizability to other ethnic groups and underscoring the importance of validation in more diverse populations such as East Asian populations.

## Conclusions

In summary, our study indicates that higher adherence to the DASH diet was modestly associated with a reduced risk of overall MSK disorders in European populations. In disease-specific analyses, protective associations were observed for most of the five specific conditions examined, particularly OA, low back pain, and neck pain, whereas associations with RA and gout were less consistent. The protective association was broadly consistent across most genetic risk strata, although a potential interaction was noted for RA. These findings suggest that promoting the DASH diet may confer population-level benefits for musculoskeletal health, although the modest effect sizes and heterogeneity across disease types should be considered when interpreting the results.

## Supplementary Information

Supplementary Material 1.

## Data Availability

The genetic and phenotypic UK Biobank data are available on application to the UK Biobank to any researcher worldwide (www.ukbiobank.ac.uk).

## Abbreviations

DASH: Dietary Approaches to Stop Hypertension
MSK: Musculoskeletal
PRS: Poly-genetic risk scores
OA: Osteoarthritis
RA: Rheumatoid arthritis
GWAS: Genome-wide association studies
SNPs: Single nucleotide polymorphisms
UKB: UK Biobank
TDI: Townsend deprivation Index
